# The Emission of VOCs and CO from Heated Tobacco Products, Electronic Cigarettes, and Conventional Cigarettes, and Their Health Risk

**DOI:** 10.3390/toxics10010008

**Published:** 2021-12-28

**Authors:** Fengju Lu, Miao Yu, Chaoxian Chen, Lijun Liu, Peng Zhao, Boxiong Shen, Ran Sun

**Affiliations:** 1 School of Chemical Engineering, Hebei University of Technology, Tianjin 300401, China; lufengjuhebut@163.com; 2 Tianjin Key Laboratory of Energy Utilization and Pollutant Control, School of Energy and Environmental Engineering, Hebei University of Technology, Tianjin 300401, China; ymiao1126@163.com (M.Y.); llj123edu@163.com (L.L.); zhaopeng1162@126.com (P.Z.); sryhc2020@163.com (R.S.); 3 Research & Development Department, Shenzhen YouMe Information Technology Co., Ltd., Shenzhen 518000, China; freddychen@iyoume.com

**Keywords:** HTPs, e-cigarettes, cigarettes, VOCs, CO, health risks

## Abstract

The differences in aerosol composition between new tobacco types (heated tobacco products and electronic cigarettes) and conventional cigarettes have not been systematically studied. In this study, the emissions of volatile organic compounds (VOCs), carbon monoxide (CO), nicotine, and tar from heated tobacco products (HTPs), electronic cigarettes (e-cigarettes) and conventional cigarettes were compared, and their health risks were evaluated by applying the same smoking regime and a loss mechanism of smoking. Twenty VOCs were identified in aerosols from HTPs, 18 VOCs were identified in aerosols from e-cigarettes, and 97 VOCs were identified in aerosols from cigarettes by GC–MS and HPLC analysis. The concentrations of total VOCs (TVOCs) emitted by the three types of tobacco products decreased as follows: e-cigarettes (795.4 mg/100 puffs) > cigarettes (83.29 mg/100 puffs) > HTPs (15.65 mg/100 puffs). The nicotine content was 24.63 ± 2.25 mg/100 puffs for e-cigarettes, 22.94 ± 0.03 mg/100 puffs for cigarettes, and 8.817 ± 0.500 mg/100 puffs for HTPs. When using cigarettes of the same brand, the mass concentrations of VOCs, tar, and CO emitted by HTPs were approximately 81.2%, 95.9%, and 97.5%, respectively, lower than the amounts emitted by cigarettes. The health risk results demonstrated that the noncarcinogenic risk of the three types of tobacco products decreased as follows: cigarettes (3609.05) > HTPs (2449.70) > acceptable level (1) > e-cigarettes (0.91). The lifetime cancer risk (*LCR*) decreased as follows: cigarettes (2.99 × 10^−4^) > HTPs (9.92 × 10^−5^) > e-cigarettes (4.80 × 10^−5^) > acceptable level (10^−6^). In general, HTPs and e-cigarettes were less harmful than cigarettes when the emission of VOCs and CO was considered.

## 1. Introduction

### 1.1. Background

Exposure to volatile organic compounds (VOCs), polycyclic aromatic hydrocarbons (PAHs), metal elements, carbon monoxide (CO), and particulate matter (PM) from tobacco combustion processes [1,2,3,4] will increase the occurrence of lung cancer, cardiovascular and respiratory problems, chronic obstruction, neurotoxicity, and pulmonary diseases in humans [5,6]. Each year, tobacco kills more than 8 million people worldwide. Over 7 million of those deaths are the result of direct tobacco use, and 65,000 children die from illnesses attributable to secondhand smoke [7]. To replace cigarettes and reduce their toxic effects, new cigarette types, such as heated tobacco products (HTPs) and e-cigarettes, have been introduced into the market. To the best of our knowledge, studies have mainly focused on the emission characteristics of toxicants from a single product or on a comparison between two types of products. For instance, the toxic elements (mercury, chromium, cadmium, and lead), VOCs, carbonyl compounds, and PAHs in main- and side-stream smoke of cigarettes have been analysed [1,2,3,4,8]. The research results of Pack et al., indicated that CO and VOCs were the main emissions from cigarettes [1,2]. Flora and Geiss et al. [9,10,11] studied the characteristics of VOCs, PM, metals, radicals, and nitrosamines from e-cigarette formulations and aerosols. Savareear, Mitova, and Mottier et al. [12,13,14] carried out a series of experiments to compare the composition of the vapour phase from cigarettes and HTPs and found that all quantified concentrations of compounds in cigarette smoke were higher than those in HTP aerosols. Kim et al., assessed the changes in the concentration of six carbonyl compounds in the aerosols of HTPs and electronic cigarettes with increasing heating temperatures (25–470 °C) [15]. Forster et al. compared the levels of potentially harmful constituents (HPHCs) in mainstream emissions from IQOS and Glo (two typical HTPs) [16,17,18,19]. Current studies have mostly relied on smoking machines to complete the smoking experiment [2,9,14,20,21], but machine smoking does not necessarily reflect how people actually use the products [22]. Different researchers have used different smoking protocols to answer different questions and have shown that more intense smoking leads to higher yields of smoke-related chemicals [20,21]. The varying VOCs and CO results from different types of tobacco products have been obtained under different smoking regimes (puff volume, puff interval, and puff duration), sampling methods (tenax/sulficarb sorbents, solvent absorption, Tedlar bags, etc.) and analysis methods (analytical instrument, chromatographic column, temperature program, etc.). The experimental results obtained by previous researchers cannot be used to accurately compare the toxicity levels of these three types of tobacco products. Currently, the reason for the discrepancies in the emission characteristics and toxicity levels from harmful substances in mainstream smoke released by HTPs, e-cigarettes, and cigarettes remains unclear.

### 1.2. Aim of the Study

The main aim of this study was to comprehensively compare the difference in the composition of aerosols emitted by HTPs, e-cigarettes, and cigarettes under the same smoking regimes, sampling methods, and analysis methods, and to evaluate the noncarcinogenic and carcinogenic health risks of VOCs and CO.

## 2. Material and Methods

### 2.1. Products Used in the Study

The main parameters of the e-cigarette products were as follows: tank capacity 1.5–3 mL, coil resistance 0.5–1.2 Ω, max output 10–60 W, working voltage 3.7–4.2 V, and battery capacity 350–1500 mAh [23]. The main parameters of the tobacco product directive certified e-cigarette (Suorin Air Plus) were as follows: tank capacity 2 mL, coil resistance 0.7 Ω, max output 22 W, working voltage 3.3–4.2 V, and battery capacity 930 mAh. This product has a built-in air pressure sensor, which automatically starts when pumping. More than 1 million units are sold per year, and this brand ranked among the top 10 in the US vape pod market from 2016 to 2020 [24,25]. The E-cigarette liquid (also called e-liquid) was produced by Shenzhen Boton Flavor Co., Ltd. and was composed of propylene glycol, glycerine, salt nicotine (20 mg/mL) and Passiflora edulis Sims flavours. The HTPs (YouMe-2) used in this study can heat cigarettes, and a cigarette filter is used as the smoke filter in this product. The YouMe-2 includes a three-section heating system and a rechargeable battery. The heating temperature of the HTPs is approximately 270 °C, and a single use reaches up to 14 puffs. The e-cigarettes and HTPs were purchased from the Shenzhen YouMe Network Technology Co., Ltd., Shenzhen, China.

**Figure 1:**
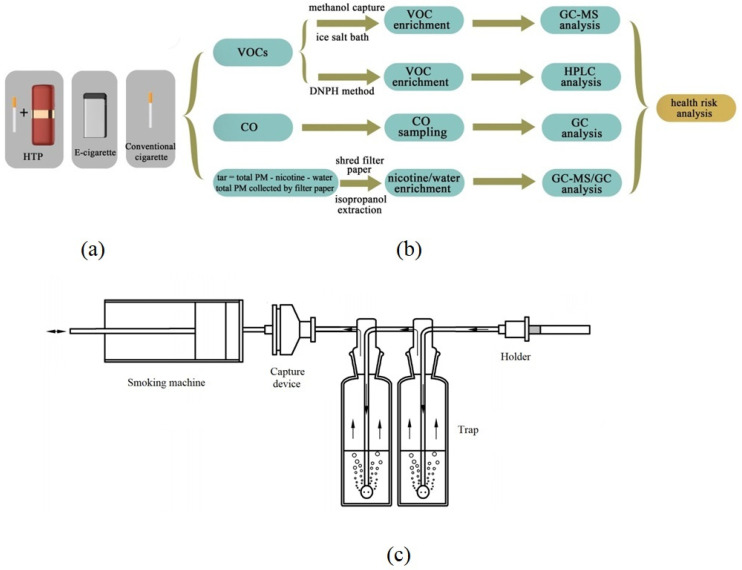
(**a**) Types of tobacco products used in this research; (**b**) Flow chart of the sampling procedure; (**c**) Device for mainstream aerosol sampling.

The content ranges of nicotine, tar, and CO from Chinese conventional cigarettes are 0.8–1.3 mg/cigarette, 9–12 mg/cigarette, and 8–12 mg/cigarette, respectively. The cigarettes (from Yunnan Province, China) selected in this study contained 1.1 mg nicotine per cigarette, 10 mg tar per cigarette, and 12 mg carbon monoxide per cigarette. This brand of cigarettes were typical Virginia-type cigarettes with cellulose acetate filters in China, and the length and diameter of each cigarette used in this study was 84 mm and 8 mm, respectively. The average weight of the tobacco leaves from each cigarette was 650 mg. The average puff count was 12 puffs/cigarette. According to the Chinese Cigarette Website https://www.cnxiangyan.com/pinpai/ (accessed on 5 June 2021), this cigarette brand ranked among the top three in the Chinese market in terms of market share from 2017 to 2020 and is representative of Chinese cigarettes. To simulate a realistic smoking scene for a smoker, we chose Chinese cigarettes, not 3R4F cigarettes (tobacco for scientific research developed by the University of Kentucky). Notably, cigarettes of the same brand were selected for comparing the emission levels of the heating method and burning method. The products are listed in Figure 1a.

### 2.2. Smoking Conditions

The experimental device is displayed in Figure 1c. The smoking procedure was performed by a multifunctional smoking machine (HW-Y102) purchased from Dongguan Hongwei Test Equipment Co., Ltd., China. The capacity range of the smoking machine was 0–100 mL, and the precision of puff volume and time were ±0.01 mL and ±0.01 s, respectively. The puff volume and puff interval of cigarettes and e-cigarettes published by ISO 3308, Health Canada Intense (HCI), CRM81 and Flora et al. [9,11,26,27] are displayed in Table S1. ISO 3308 is the intense regulatory protocol for cigarettes cited by most studies, which is convenient for comparisons among different cigarettes; they are not appropriate as the basis for determining the amount of smoke yields inhaled by actual smokers [1]. Therefore, HCI methods have been introduced to replace ISO methods. To compare the emission characteristics of VOCs/non-VOCs from different types of tobacco products under the same smoking regimes, the cigarette smoking and e-cigarette puffing regimes were executed uniformly according to HCI and CRM81 in this study (puff volume 55 mL, duration time 3 s, interval time 30 s). Different types of tobacco products were placed on the holder and connected to the silicone tube of the multifunctional smoking machine. After setting all the parameters, the smoking machine was started. To increase the method sensitivity, the total puff number for the three types of tobacco products was set to 100 puffs with reference to the research of Flora et al. [9]. The mainstream aerosols emitted by the different types of tobacco products flowed into the two traps successively and were absorbed by the trapping solution. The dimensions of the experimental room were 5.8 m (L) × 3.7 m (W) × 2.6 m (H). The thermohydrometric conditions of the room were maintained at a temperature of 22 ± 2 °C and a relative humidity of 60 ± 5% by a household air conditioner unit during the experiment [9]. The room used for the experiment was empty, and there were no other sources of pollution. The effective airtight windows and doors were closed to avoid pollution from the outdoor environment. The background VOCs, nicotine, tar, and CO were sampled and analysed without a nicotine source.

### 2.3. Aerosol Analysis

#### 2.3.1. VOCs in Smoke and Aerosols

The VOCs were analysed by a Shimadzu GC–MS system (Shimadzu GCMS-QP 2020, Kyoto, Japan) and Agilent HPLC system (Agilent Technologies 1260 Infinity II, Böblingen, Germany). The sampling and analysis method was based on the standard methods in the CORESTA Recommended Method N^o^ 70 and 74. [28,29,30]; the details are shown in the supporting information (S2.1).

#### 2.3.2. Nicotine in Smoke and Aerosols

Before smoking, the two traps (shown in Figure 1c) were removed, and then a capture device equipped with glass fibre filter paper was used to collect nicotine in the smoke and aerosols. The details of the sampling and analysis procedure are shown in the supporting information (S2.2).

#### 2.3.3. Tar in Smoke and Aerosols

The tar content was determined by deducting the water and nicotine contents from the total PM [31]. The total PM was collected with a glass fibre filter, and the mass difference in the glass fibre filter was calculated by electronic balance after collecting the smoke or aerosols. Nicotine in the smoke and aerosols was analysed according to S2.2. The water was analysed based on the method of GB/T 23203.1 [32] (shown in S2.3).

#### 2.3.4. CO in Smoke and Aerosols

The CO in smoke and aerosols was collected with a PTFE sampling bag (2 L). Before smoking, the two traps and the capture device (shown in Figure 1c) were removed, and then the sampling bag was connected to the exhaust vent of the smoking machine to collect the carbon monoxide emitted by the three types of tobacco products. According to the standard method [33], the bag was cleaned and emptied using clean air before each experiment. Details are shown in the supporting information (S2.4). The flow chart of sampling (VOCs, nicotine, water, tar, and CO) is shown in Figure 1b.

The sampling and analysis of VOCs, nicotine, tar, and CO released by each type of tobacco were performed three times with repeated experiments. To avoid the mutual influences of sampling, the test of each tobacco product was discontinuous. Before each sampling experiment, the experimental room was cleaned and ventilated vigorously to ensure the accuracy of the experimental results. The background VOCs, nicotine, tar, and CO concentrations were below the detection limit.

### 2.4. Risk Assessment of Different Types of Tobacco Products

In real smokers, part of the smoke that is kept in the smoker’s mouth leaks out with the removal of the cigarette butt from the mouth, together with an additional amount that can be voluntarily or involuntarily discharged prior to inhalation. This portion is called mouth-spill (*MS*). Furthermore, toxicants in the upper respiratory tract are exhaled and not fully absorbed; this part is known as respiratory retention (*RR*). Considering the loss mechanism in which smoke spills from the mouth and is exhaled from the respiratory tract in the smoking process, a method related to *MS* and *RR* was used to optimise the exposure model from mainstream smoke [1,34]. In this study, 21 toxicants (shown in Table S8) were used to determine the health risk of different types of tobacco products. According to Equation (1) [1], the concentration of 21 toxicants in mainstream smoke (*C_i_*) was converted to systemic smoker uptake (*U_i_*), mg/puff. The *RR* values are listed in Table S8, and *MS* was estimated to be 30% based on the result of St. Charles’s research [34]. The *U_i_* results are listed in Table S9.

(1) $$U_{i}=C_{i}\times RR_{i}\times \left(1-MS\right)$$

In this study, adult male smokers (Chinese) were chosen to evaluate the human health risk from three types of tobacco products according to the lifetime cancer risk (*LCR*) and hazard index (*HI*) [35,36].

(2) $$CDI=U_{i}\times IR\times EF\times ED/\left(365\times BW\times AT\right)$$

where *CDI* is the exposure dose of constituent *i* emitted by different types of tobacco products (mg/(kg d)), and the results are listed in Table S9; *IR* is the intake rate of cigarettes (puff/day); *EF* represents the exposure frequency during an entire year (365 days/year); *ED* represents the exposure duration for smoking (54 years, average lifetime minus 18 years); *BW* is the human body weight (62.7 kg) [37]; and *AT* is the time period over which the dose is averaged [36] (noncarcinogenic *AT* = *ED*, carcinogenic *AT* = lifetime), assuming an average lifetime of 72 years (male) [37].

For most smokers, the main purpose of smoking is to relieve addiction, and to our knowledge, cigarettes have the highest market share. The average daily exposure of cigarettes was 15.2 cig/day [38], each cigarette could be used to smoke 12 puffs [2], and the concentration of nicotine was 0.229 mg/puff (tested by this research). Therefore, the average daily exposure dose of nicotine for most smokers was 41.85 mg/day. According to the equal exposure dose of nicotine, the health risks of the three types of tobacco products were calculated.

HI, used as the noncarcinogenic health risk indicator of smoking, was determined according to Equation (3) [35,37].

(3) $$HI=CDI_{i}/RFD_{i}$$

(4) HIs=∑HI

where *HI* is the noncarcinogenic hazard index of constituent *i*; *RFD* is the noncarcinogenic reference dose for toxic pollutants (mg/kg-day), shown in Table S8; and *HIs* is the sum of the hazard index values for all constituents in the aerosols of different types of tobacco products. For constituent *i*, a value of *HI* less than 1 indicates that the health risk is acceptable, while values of 1 or greater are hazardous [39,40].

(5) LCR=CDI×SF

where *SF* is the cancer slope factor for constituent *i*, (mg/kg-day)^−1^, displayed in Table S8. For cancer risk, the acceptable cancer risk is 10^−6^, and a higher *LCR* value indicates a more hazardous pollutant [41].

### 2.5. Statistical Analysis

The experimental results are described as mean ± standard deviation (SD). Statistical analysis was performed with GraphPad Prism 8.0. ANOVA was used to understand the concentration relations of the total VOCs released by cigarettes, HTPs, and e-cigarettes. The t test was used to analyse the concentration relations of the tar, water, and CO released from cigarettes under different smoking methods (the heating method and burning method). The *p*-values of ANOVA and t test were less than 0.05, indicating that the data analysis was meaningful.

## 3. Results

### 3.1. VOCs Emission

#### 3.1.1. GC-MS and HPLC Chromatographic Analysis of VOCs

**Figure 2:**
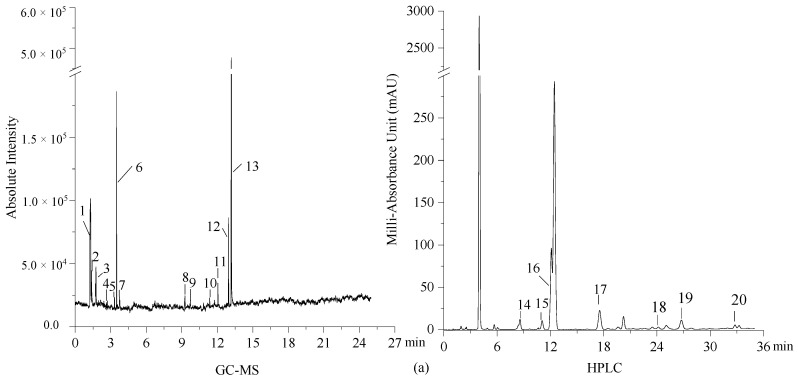

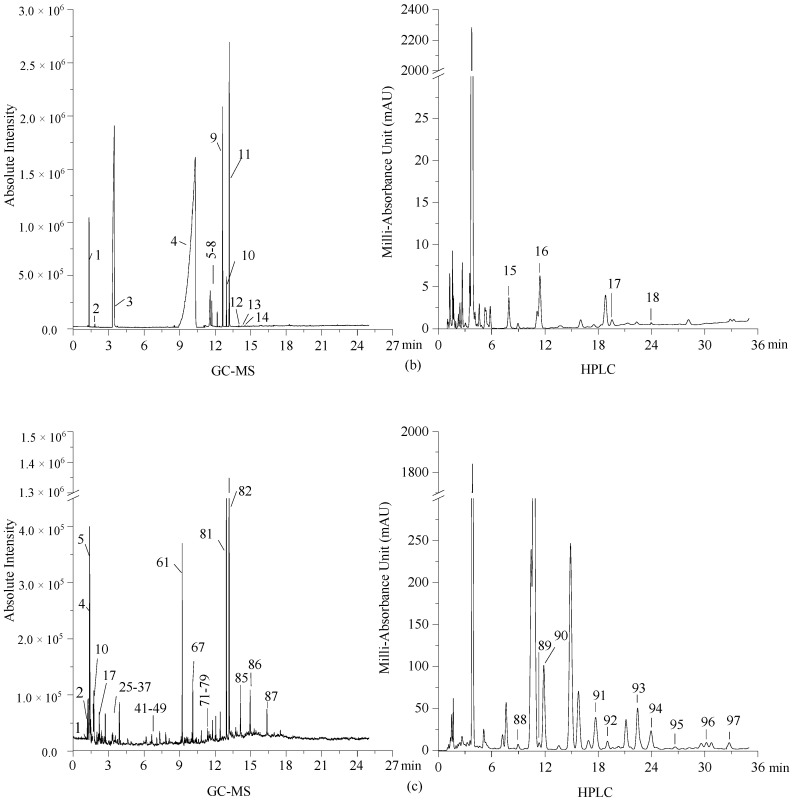
GC-MS and HPLC chromatograms of the VOCs emitted by HTPs (**a**), e-cigarettes (**b**) and cigarettes (**c**).

The number in Figure 2 represents the VOCs tested in the mainstream aerosols of the three types of tobacco products. As expected, a strong contrast was observed among HTPs, e-cigarettes, and cigarettes. The GC-MS peaks of VOCs emitted by cigarettes and HTPs within 1 to 2 min and 12 to 14 min were similar. Twenty VOCs existed in the mainstream aerosols of HTPs, 18 VOCs were present in the aerosols from e-cigarettes, and 97 VOCs were emitted from cigarettes (listed in Tables S2–S4). Nineteen VOCs, namely 2,2-dimethoxybutane, triacetin, formaldehyde, acetaldehyde, propyl aldehyde, acrolein, butyl carbamate, methyl acetate, 5-hydroxymethylfurfural, nicotine, glycerine, propylene glycol, 3-methylfuran, 2,5-dimethylfuran, pyranone, acetone, butanone, benzaldehyde, and tolualdehyde were detected in the aerosols of HTPs and cigarettes. Seven VOCs (triacetin, formaldehyde, acetaldehyde, butanone, nicotine, glycerine, and propylene glycol) all existed in the aerosols of all three types of tobacco products. Notably, glycerine is easily released from cigarettes, and propylene glycol is released from HTPs.

#### 3.1.2. Analysis of the Types and Concentrations of VOCs

**Figure 3:**
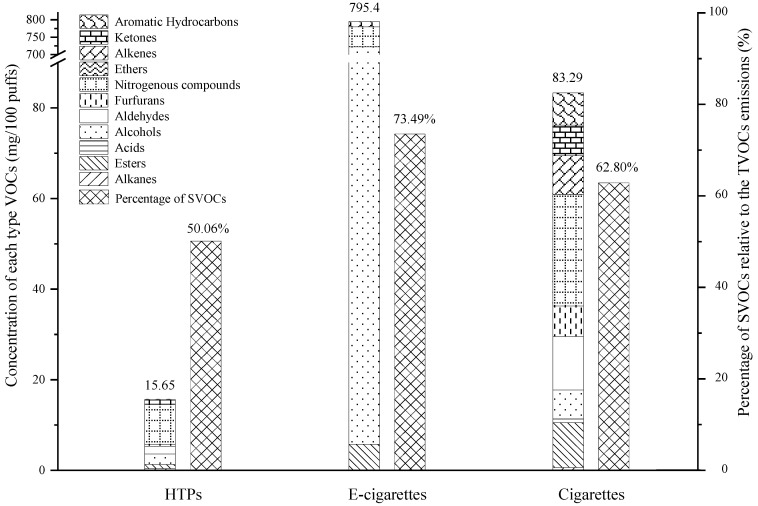
Types of VOCs produced by three types of tobacco products and the percentage of semi-VOCs (SVOCs) relative to the total VOCs (TVOCs) emission.

As shown in Figure 3, the types, and concentrations of VOCs in the mainstream aerosols of HTPs, cigarettes, and e-cigarettes were clearly distinct. The ANOVA results of total VOCs (TVOCs) show statistically significant differences among the groups (ANOVA test *p* < 0.0001), particularly for e-cigarettes and HTPs (ANOVA, *p* < 0.0001), and e-cigarettes and cigarettes (ANOVA, *p* < 0.0001). The total VOCs (TVOCs) content emitted by e-cigarettes was the highest (795.4 mg/100 puffs), which was 50.8-fold that of HTPs and 9.5-fold that of cigarettes. There were six types of VOCs in e-cigarette aerosols, which only contained alcohols, nitrogenous compounds, benzoic acid, esters, aldehydes, and ketones, accounting for 90.12%, 7.22%, 1.92%, 0.73%, 0.01%, and < 0.01%, respectively. Eight types of VOCs existed in the mainstream aerosols of HTPs, which contained nitrogenous compounds, alcohols, aldehydes, ketones, esters, alkanes, furfurans, and aromatic hydrocarbons, accounting for 56.5%, 14.6%, 11.0%, 5.9%, 5.8%, 2.5%, 2.5%, and 1.2%, respectively. Eleven types of VOCs existed in the mainstream aerosols of cigarettes, which contained nitrogenous compounds, aldehydes, esters, alkenes, aromatic hydrocarbons, ketones, alcohols, furfurans, acids, alkanes, and ethers, accounting for 29.52%, 18.40%, 11.92%, 10.27%, 8.54%, 8.13%, 7.64%, 3.79%, 0.98%, 0.77%, and 0.03%, respectively. For e-cigarettes, the concentration of alcohols was the highest. For HTPs and cigarettes, the concentration of nitrogenous compounds was the highest. The nicotine content of e-cigarettes and cigarettes was approximately 2.8-fold and 2.6-fold higher than that of HTPs (shown in Tables S5–S7). Semi-VOCs (SVOCs) have a boiling point in the range of 240–400 °C and have the advantages of poor degradability and strong adsorption; SVOCs enter the human body through the lungs or skin and cause various diseases, especially asthma and allergies [42]. The SVOCs released from smoking should be given more attention. As shown in Figure 3, the mass percentages of SVOCs released from e-cigarettes with respect to TVOCs were the highest, followed by those of cigarettes and HTPs.

The formaldehyde and acetaldehyde contents in the mainstream aerosols of e-cigarettes measured in this study were 1.13 ± 0.22 × 10^−2^ and 5.64 ± 1.09 × 10^−3^ µg/mL (converted at 55 mL per puff), which coincide with the results of Stephens et al. (8.07 × 10^−3^ and 4.41 × 10^−3^ µg/mL) [43]. Ethyl acetate, ethanol, DL-menthol, and benzoic acid are the main additives in e-liquid formulations. Nicotine, propylene glycol, and 5-hydroxymethylfurfural were the main emissions from HTPs, accounting for 57%, 14%, and 8%, respectively. The number of aromatic hydrocarbons emitted by cigarettes was 20, with a concentration of 7.112 mg/100 puffs, accounting for 8.5% of the total emission concentration. Benzene, toluene, ethylbenzene, p-xylene, styrene, naphthalene, 1,2,3-trimethylbenzene, and 1,2,4-trimethylbenzene were much more easily produced by cigarettes than HTPs or e-cigarettes. Although the emission content of aromatic hydrocarbons was not the highest, its toxicity to the human body is higher than that of other VOCs, especially benzene, toluene, ethylbenzene, and xylene (BTEX). It has been reported that high concentrations of BTEX can damage the lungs and nervous system and even cause cancer [35]. Xie et al. analysed the mainstream smoke constituent yields from twenty Chinese cigarette brands. The emission concentrations of nicotine, acrolein, acetone, toluene, benzene, isoprene, and CO were mainly 18–22 mg/100 puffs, 0.7–1.0 mg/100 puffs, 3.0–4.4 mg/100 puffs, 0.9–1.5 mg/100 puffs, 0.7–1.0 mg/100 puffs, 4.3–7.9 mg/100 puffs, and 22–27 mg/cig, respectively [2]. The results of this research matched those of Xie et al.

#### 3.1.3. Variations in the Proportion of Each Type of VOCs between the Heating Method and the Burning Method

**Figure 4:**
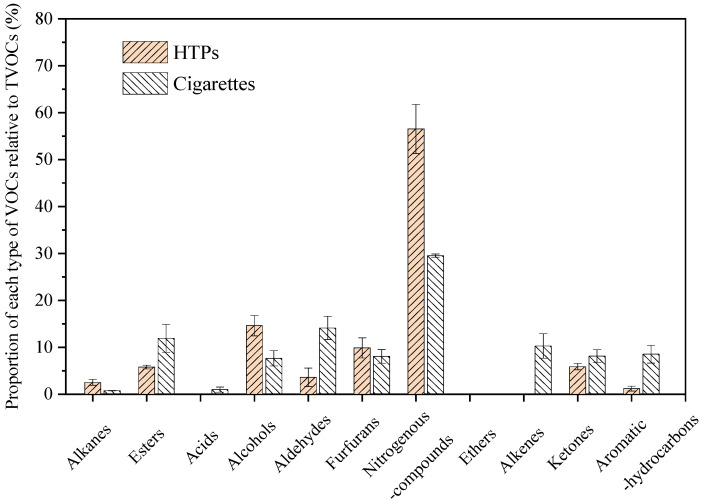
Proportion of each type of VOC relative to the TVOCs emitted by the heating method and burning method.

In terms of the emission intensity and types of VOCs, the heating method for conventional tobacco leaves changed the emission characteristics of VOCs. The proportions of alkanes, alcohols, furfural, and nitrogenous compounds with respect to the TVOCs emitted by the heating method were considerably higher than those emitted by the conventional burning method, but esters, aldehydes, kenton, and aromatic hydrocarbons exhibited the opposite trend (shown in Figure 4). Even acids, ethers, and alkenes were not found in the aerosols of the HTPs. The types and concentrations of VOCs have a significant influence on the aroma of tobacco smoke. Aldehydes, ketones, furfuran, and nitrogenous heterocyclic compounds release burnt, sweet, and baking aromas. Aromatic compounds and phenols have the characteristics of smoky herbaceous, balsamic, and spicy notes [44]. Hydroxytoluene, benzoic acid, and neophytadiene are important constituents that determine the aroma of cigarettes [45]. Ester compounds have good effects on the aroma of mainstream smoke. Some low-molecular-weight fatty acids have flavours of fruit, wine, fat, or wax, and high-molecular-weight fatty acids produce pure and mild aromas [44]. The heating method for tobacco leaves can increase the sweet and baking aromas and reduce the herbaceous smoky and spicy notes.

### 3.2. Non-VOC Emissions

**Figure 5:**
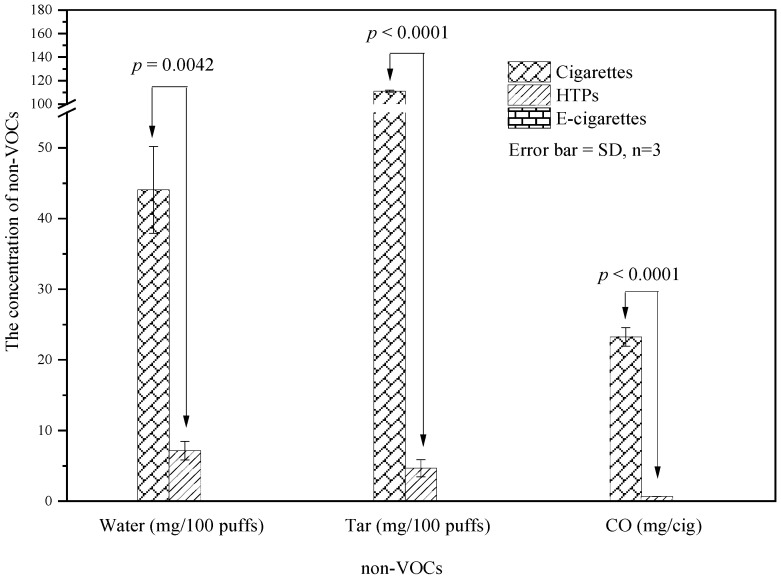
Concentrations of non-VOCs emitted from different types of tobacco products.

Non-VOCs, including water, tar, and CO, in the aerosols of e-cigarettes were below the quantifiable limits. The *t* test results of water, tar, and CO show statistically significant differences between HTPs and cigarettes (*t* test *p* < 0.05). The content of tar emitted by cigarettes was 24.14-fold that of HTPs (shown in Figure 5). It was reported that the risk of death and oral, pharyngeal, and oesophageal cancer for high-tar cigarette smokers was higher than that for low-tar cigarette smokers [46]. Long-term exposure to low concentrations of CO increases the general risk and affects the cardiovascular and respiratory systems. The emission of CO from cigarettes in this study reached 23.24 ± 1.31 mg/cig, which is obviously harmful to smokers. The adverse effects of CO for infants, foetuses, and pregnant women are greater than those for other people [6], and these groups should avoid passive smoke. For the same tobacco leaves, the amounts of nicotine, water, tar, and CO emitted by the cigarette burning method were considerably higher than those emitted by the heating method.

### 3.3. Comparative Analysis of Common Pollutants Released from Different Types of Tobacco Products and 3R4F

The scientific research cigarette developed by the University of Kentucky is called 3R4F. Common pollutants released from different types of tobacco products and 3R4F were comparatively analysed. As shown in Table 1, the concentration of CO released by 3R4F was 65.1 to 70.2-fold that of HTPs and 1.6 to 1.8-fold that of cigarettes. The concentrations of all common VOCs released by HTPs were lower those for 3R4F. For e-cigarettes, the concentrations of formaldehyde and acetaldehyde were lower than that of 3R4F, but the propylene glycol and glycerol were the opposite. Except for styrene, acetaldehyde, propylene glycol, and glycerol, the concentrations of the remaining VOCs released by cigarettes were close to that of 3R4F, especially nicotine. In general, the common pollutants released by cigarettes are highly similar to 3R4F, followed by HTPs, and e-cigarettes have the lowest similarity.

### 3.4. Human Health Risk Characterization for Different Types of Tobacco Products

**Figure 6:**
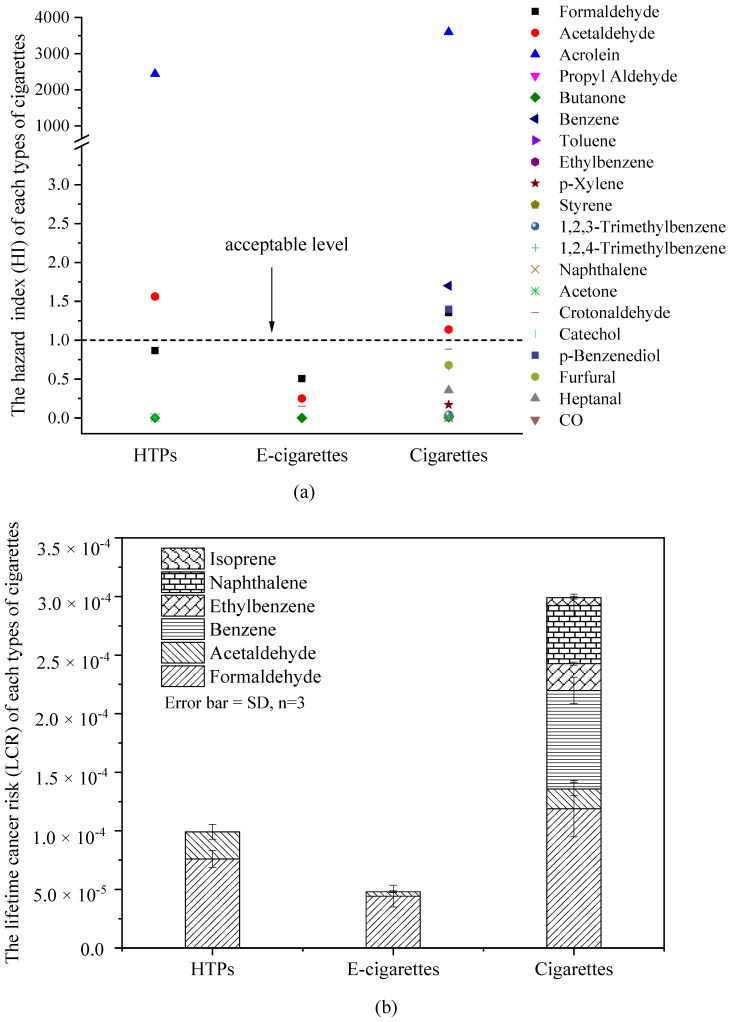
Calculated *HI* (**a**) and *LCR* (**b**) of constituents in different types of tobacco products.

The *HI* and *LCR* were calculated for 21 constituents with available toxicity data from the USEPA, CalEPA, and ATSDR [1,48], and the results are presented in Figure 6. As shown in Figure 6a, seven constituents were used to evaluate the hazard index of HTPs, and the sum of HI (HIs) was 2449.70. The HIs of the four constituents emitted by e-cigarettes was 0.91. The HI of cigarettes was assessed by considering 20 constituents. The HIs of cigarettes reached 3609.05. The above results indicate that only the noncarcinogenic health risk of e-cigarettes was acceptable. Notably, the HI values of acrolein from HTPs and cigarettes were 2442 and 3595, respectively. Haussmann et al. summarised the noncarcinogenic index of harmful substances in mainstream cigarette smoke and found that the effect of acrolein on the noncarcinogenic index was ranked as the top priority [49]. Acrolein is classified as a category III carcinogen by the WHO, and as a contaminant, it may cause adverse effects in the central and peripheral nervous systems, respiratory tract, and various cardiovascular organs [22,50]. The risk of acrolein emitted by cigarettes for human health cannot be ignored.

As shown in Figure 6b, the total *LCR* of conventional cigarettes was the highest (2.99 × 10^−4^), which was 3.01-fold that of HTPs and 6.23-fold that of e-cigarettes. The contribution rate of formaldehyde to the health risk from smoking was the highest, 76.6% for HTPs, 92.2% for e-cigarettes, and 39.7% for conventional cigarettes. The cancer risks of formaldehyde, acetaldehyde, isoprene, naphthalene, ethylbenzene, and benzene emitted by the three types of tobacco exceeded the acceptable level of 1 × 10^−^^6^.

## 4. Discussion

Instead of using ISO 3308, HCI smoking regimes were considered as the basis for determining the cigarette smoke yields inhaled by actual smokers. CRM 81 is the standardised smoking method certified by CORESTA to measure the constituent contents of e-cigarettes. In this study, to accurately compare the differences in pollutant emissions from HTPs, e-cigarettes, and cigarettes, we took into account two smoking regimes (CHI and CRM 81) and used a smoking method with a puff volume of 55 mL, a duration of 3 s, and an interval of 30 s, but in fact, people may tend to puff differently on cigarettes and e-cigarettes. In contrast to the result of Xie et al. [2], a loss mechanism of smoking considering MS and the RR models proposed by Charles et al. [34] was introduced in this study to optimise the mainstream exposure model to more accurately estimate the health risk from smoking. The exposure factors included the exposure concentration of toxicants (*Ci*), intake rate of cigarettes (*IR*), exposure frequency (*EF*), exposure duration (*ED*), human body weight (*BW*), and averaging time (*AT*), which are directly related to smoker exposure characteristics [36]. The classical exposure model reported by the USEPA [36] considers all the above exposure factors. Stephens et al. [43] established a new aggregate model to estimate the *LCR* of combustible tobacco based only on *Ci*, exposure time (*ET*), and *IR*. Considering that the main purpose of smoking is to relieve addiction, nicotine at 41.85 mg/day is the basis for the risk assessment in the classical exposure model. Compared to the results of Stephens et al., combined with loss mechanism (*MS* and *RR*) and a set amount of nicotine, the classical exposure model used in this study more comprehensively evaluate the risk level of smoking.

Twenty-one compounds, including aldehydes (formaldehyde, acetaldehyde, acrolein, propyl aldehyde, heptanal, and crotonaldehyde), ketones (butanone and acetone), aromatic hydrocarbons (benzene, toluene, ethylbenzene, *p*-xylene, naphthalene, p-benzenediol, catechol, styrene, 1,2,3-trimethylbenzene, and 1,2,4-trimethylbenzene), alkenes (isoprene), furans (furfural), and CO were selected to evaluate their *HI* and *LCR* for oral exposure. The *RFD* and *SF* values of other VOCs in the aerosols of cigarettes are unavailable, and the physicochemical effects of these components are not clear. Therefore, this method of risk assessment has limitations [22,49]. However, in addition to excluding the effects of *RFD* and *SF* values, the most important factor has been overlooked, specifically, exposure to secondhand smoke. Actual smokers inhale not only mainstream smoke but also secondhand smoke from smoking. It has been reported that in China, the smoking rate of males is 50.5%, and that of females is 2.6% [51]. We chose adult male smokers (Chinese) to evaluate the human health risk for different types of tobacco products, and the results of the risk evaluation in this study are representative.

The results show that as the main solvent components of e-liquids, glycerine and propylene glycol had the highest concentrations in e-cigarettes (707.6 mg/100 puffs), accounting for 89% of the total emission concentration. Ethyl acetate, triacetin, DL-menthol, and benzoic acid are the main flavors in e-liquid, accounting for 2.6% of the total emission concentration. E-liquid solvents and flavour agents are generally regarded as safe for oral consumption, but biological systems are complex, and the effects of their aerosolization and subsequent inhalation are not clearly understood [52]. Referring to current toxicological data, the hazard index, and the lifetime cancer risk of VOCs released from E-cigarettes, the mathematical calculation results were 0.91 and 4.80 × 10^−5^. Sood and Phillip et al., found that chronic exposure to glycerine and propylene glycol may result in impaired lung function [52,53]. Common flavour agents can also cause occupational asthma [53]. Therefore, e-liquid solvents and flavour agents may be potential factors that increase the risk of e-cigarettes.

The parameter ranges of coil resistance, max output, and working voltage for e-cigarette products sold in the market were 0.5–1.2 Ω, 10–60 W, and 3.7–4.2 V. Gillman et al. found that e-cigarette devices produced more aerosol with an increasing amount of power applied to the coil [54]. The coil resistance, max output, and working voltage of the e-cigarette devices in this study were 0.7 Ω, 22 W, and 3.3–4.2 V; therefore, the results of pollutant concentrations and health assessments only represent medium-power e-cigarettes.

## 5. Conclusions

E-cigarettes and HTPs have become popular among smokers because they are considered to be a less harmful and toxic alternative to tobacco cigarettes. However, the emission characteristics and toxicity levels of toxicants emitted from new types of tobacco products and cigarettes have not been systematically compared. It is worth detecting and analysing the emission characteristics of tar, nicotine, CO, and VOCs, the main components emitted from smoking, from HTP, e-cigarettes, and cigarettes. In this study, we systematically compared the chemical components of mainstream aerosols from the above three products with a carefully designed smoking method and evaluated the noncarcinogenic and carcinogenic health risks via *HI* and *LCR* under the MS and RR models. Fewer than 20 VOCs were found in the aerosols of HTPs and e-cigarettes, but more than 90 VOCs were present in the mainstream smoke of cigarettes. The results show that the total concentration of VOCs emitted by e-cigarettes was the highest (795.4 mg/100 puffs), which was 50.8-fold and 9.5-fold higher than that emitted by HTPs and cigarettes, respectively. For the same cigarettes, the emission concentrations of VOCs, tar, and CO for the heating method were considerably reduced by approximately 81.2%, 95.9%, and 97.5% when compared to the burning method. The type and concentration of VOCs affect the aroma of HTPs and cigarettes. The health risk results for the different types of tobacco products reveal that only the noncarcinogenic health risk of e-cigarettes was acceptable (0.91). The carcinogenic health risk of cigarettes was 2.99 × 10^−4^, which was 3.01-fold that of HTPs and 6.23-fold that of e-cigarettes, all of which exceeded the acceptable level of 1 × 10^−6^. In general, HTPs and e-cigarettes are less harmful than cigarettes.

## Figures and Tables

**Table 1:** The concentration of common pollutants released from different types of tobacco products and 3R4F.

Pollutants (ug/mL)	HTPs	E-Cigarettes	Cigarettes	3R4F ^a^	3R4F ^b^
CO	0.88	ND	35.2	57.3	61.8
Styrene	ND	ND	0.050	0.026	0.004
Isoprene	ND	ND	1.0	1.7	0.89
Benzene	ND	ND	0.12	0.17	0.60
Toluene	ND	ND	0.28	0.29	0.13
Catechol	ND	ND	0.22	0.18	/
Formaldehyde	0.007	0.011	0.028	0.085	/
Acetaldehyde	0.013	0.005	0.024	4.3	2.2
Acetone	0.15	ND	0.82	1.2	/
Acrolein	0.04	ND	0.17	0.26	0.11
Nicotine	1.6	4.5	4.17	3.6	4.2
Propylene glycol	0.38	30.7	0.12	0.062	/
Glycerol	0.03	98.0	0.94	4.1	/

Note: The unit (ug/mL) represents the concentration of the common pollutants per millilitre of aerosol; “ND” represents not detected; “^a^” and “^b^”represents the concentration of the common pollutants released from 3R4F according to the research by Hirn and Marcilla et al. [21,47].

## Data Availability

Not applicable.

## Supplementary Materials

The following are available online at https://www.mdpi.com/article/10.3390/toxics10010008/s1, Table S1: Standardised tobacco smoking regimes and e-cigarette puffing regimes, Table S2: Chromatogram information of the VOCs emitted by HTP, Table S3: The chromatograms information of the VOCs emitted by e-cigarettes, Table S4: The chromatograms information of the VOCs emitted by cigarettes, Table S5: The concentrations of VOCs and non-VOCs measured from HTPs, Table S6: The concentrations of VOCs and non-VOCs measured from e-cigarettes, Table S7: The concentrations of VOCs and non-VOCs measured from cigarettes, Table S8: The related parameters of the risk assessment, Table S9: The value of *U* and *CDI* for different constituents, *i*, emitted by different types of cigarette. Reference [55] is cited in the supplementary materials.
