# Supplementary material for: The Emission of VOCs and CO from Heated Tobacco Products, Electronic Cigarettes, and Conventional Cigarettes, and Their Health Risk
Source: Toxics. 2021 Dec 28;10(1):8. doi: 10.3390/toxics10010008 (PMC8781168; doi:10.3390/toxics10010008)
Supplement: Supplementary file 1 [file toxics-10-00008-s001.zip › toxics-1452429-supplementary.pdf]

toxics

Supplementary Materials: The Emission of VOCs and CO from Heated Tobacco Products, Electronic Cigarettes, and Conventional Cigarettes, and their Health Risk

Fengju Lu, Miao Yu, Chaoxian Chen, Lijun Liu, Peng Zhao, Boxiong Shen, Ran Sun

S1 Instruments and materials

Table S1. Standardised tobacco smoking regimes and e-cigarette puffing regimes.

**Condition**	**Puff volume (mL)**	**Duration time (s)**	**Approximate puff count**	**Interval time (s)**
ISO 3308 (cigarettes)	35	2	5-10/cig	60
MDPH (cigarettes)	45	2	8-15/cig	30
HCI (cigarettes)	55	2	6-14/cig	30
CRM 81(e-cigarettes)	55	3	Up to battery exhaustion	30
Flora et al.[1]	55	4	Up to battery exhaustion	30
Wagner et al.[2]	55	5	Up to battery exhaustion	30
This study	55	3	12/cig or up to battery exhaustion	30

Note: HCI, Health Canada Intense; ISO, International Organization for Standardization; MDPH, Massachusetts Department of Public Health.

S2. Aerosol analysis

S2.1. Volatile organic compounds in the smoke and aerosols

The sampling and analysis method of volatile organic compounds was based on the standards [3,4]. As a trapping agent, 40 mL of methanol was added to each trap and placed in an ice salt bath. The trapping agent capturing the VOCs was filtered by a 0.45 μm PTFE filter membrane and then analysed by a Shimadzu GC–MS system equipped with a capillary column (Rxi-5Sil MS Cap Column, 30 m × 0.25 mm × 0.25 μm). The analysis conditions of the GC–MS system were as follows: the temperature was 40 °C initially, then held for 6 min at 40 °C, which was then increased to 230 °C at 20 °C/min, and held for another 6 min at this temperature. The total flow and column flow of carrier gas (gaseous helium) were maintained at 27 mL/min and 1.5 mL/min, respectively. The injection volume of the samples and standards was 1 μL, and the split ratio of helium was 15:1. The temperature of the injection port for GC was 180 °C. The ion source temperature of the MS system and the transmission line temperature between GC and MS were both 250 °C. The mass number scan range of the MS system ranged from 35 to 500 *m*/*z*, with a scanning interval of 0.3 s.

The VOC standards were diluted to five different concentrations with HPLC-grade methanol (purity ≥ 99.99%); the correlation coefficient of all VOC standard curves (R^2) was not less than 0.999. In special circumstances, appropriate standards could not be obtained for the target compound. In these cases, secondary standards were used for quantification based on similar retention times or chemical structures. The proportion recoveries of the method ranged from 90% to 106%. The limits of detection and quantification ranged from 0.06 to 0.84 μg/100 puffs and from 0.1 to 3.0 μg/100 puffs, depending on the standards. The precision of the Shimadzu GC–MS system was 3–8%.

Considering that the VOCs (molecular weight close to the extractant) could not be qualified and quantified by GC–MS, ten kinds of aldehydes and ketones (formaldehyde, acetaldehyde, acrolein, propyl aldehyde, crotonaldehyde, methacrolein, butanone, pen-

tanal, benzaldehyde and tolualdehyde) in smoking aerosols were analysed with an Agilent HPLC system. All target components were trapped in the two traps, as per the DNPH method [5]. The trapping agent was prepared with 2,4-dinitrophenylhydrazine (DNPH) (4.5 g), acetonitrile (200 mL), and dilute phosphoric acid (10%) (20 mL), and then all the above reagents were mixed and diluted to 500 mL with ultrapure water. For each experiment, 40 mL of trapping agent was added into the two traps.

After finishing the trapping, 2 mL pyridine was mixed into each trap. The 10 ml mixture was filtered through a 0.45 μm PTFE filter membrane. The target components filtered by filter membrane were analysed with an Agilent HPLC system (Agilent Technologies 1260 Infinity II, Germany) with the elution gradient function. The instrument was equipped with a UV detector and a C18 column produced by Shimadzu (Shim-pack GIST, 150 mm × 4.6 mm × 5 μm). The mobile phase was prepared with ultrapure water, acetonitrile, tetrahydrofuran and isopropanol. The proportions of ultrapure water:acetonitrile:tetrahydrofuran:isopropanol were: A phase 63:27:9:1; B phase 40:58:1:1; and C phase 0:100:0:0. The analysis conditions of the Agilent HPLC system were set as follows: the temperature of the C18 column was set to 50 °C and the wavelength of the UV detector was 365 nm. Total flow of the mobile phase was 1.25 mL/min. The injection volumes of the samples and the standards were both 5 μL.

The qualitative aldehydes/ketones were finished by mixing standards at a concentration of 5 μg/mL. The range of recovery for aldehydes/ketones was between 90% and 105% based on this method. The limits of detection and quantitation for the method were 0.2–0.4 μg/100 puffs and 0.6–1.3 μg/100 puffs, respectively. The precision of the Agilent HPLC system was 4% to 12%, depending on the mix standards.

S2.2. Nicotine in the smoke and aerosols

Before smoking, the two traps (shown in Figure 2) were removed, and then a catcher equipped with glass fibre filter paper was used to collect nicotine in smoking aerosols. After completing the aspiration of 100 puffs, the glass fibre filter paper was taken off, and then the catcher was wiped with new glass fibre filter paper. All experimental filter papers were shredded and soaked in 40 mL isopropanol, and then the nicotine in the filter papers was extracted under 30 min machine concussions. The extracts containing nicotine were analysed according to the GC–MS method detailed in Section S2.3.1.

S2.3. Tar in the smoke and aerosols

The tar represents the total particulate matter deducted the water and nicotine [6]. The total particulate matter was collected with glass fibre filter paper, and the mass difference of the glass fibre filter paper was calculated by electronic balance after collecting the smoking aerosols. The extraction and analysis process of nicotine in the aerosols was executed according to S2.3.2. The water was analysed based on the method of GB/T 23203.1 [7].

First, the glass fibre filter papers collected the smoking aerosols were impregnated in 40 mL isopropanol, and the water in the glass fibre filter papers was extracted under 30 min machine concussions. Then, the extracts containing the water were qualitatively and quantitatively analysed using a Shimadzu GC system (GC 2010, Japan) equipped with a packed column (Porapak Q 2 m×3 m (od)) purchased from Lanzhou Institute of Chemical Physics, China. The total flow rate of carrier gas (gaseous helium) was 30 mL/min. The procedure temperature of the packed column was maintained at 170 °C. The temperature of the injection port and the detector were 250 °C. The injection volume of the samples and the water standard was 2 μL. The correlation coefficient of the water standard curves (R^2) was not less than 0.999. The percentage recoveries of the method ranged from 92% to 101%. The limits of detection and quantification for this method ranged from 1.2 to 3.0 μg/100 puffs and from 4.2 to 9.8 μg/100 puffs. In addition, the precision of the Shimadzu GC system was 2–6%.

S2.4. Carbon monoxide in the smoke and aerosols

Carbon monoxide in smoking aerosols was collected in a PTFE sampling bag (2 L). Before smoking, the two traps and the catcher (shown in Figure 2) were removed; then, the sampling bag was connected to the exhaust vent of the smoking machine to collect the carbon monoxide emitted by a cigarette. In accordance with the standards [8], the bag was cleaned with clean air and emptied before each experiment. The concentration analysis of CO was finished by GC (GC 2010 Plus, Japan) with a column (TDX-01) and a thermal conductivity detector. The injection volume of the samples and standards was 0.5 mL. The analysis procedure of GC was as follows. The temperature of the column and the injection port was 100 °C. The temperature of the thermal conductivity detector was 90 °C. Gaseous helium was used as the carrier gas, with a total flow of 30 mL/min. The CO emitted by three types of cigarettes was quantified by different concentrations of standard gas. The repeatability of sample collection was 95-105%, and the precision of the Shimadzu GC system was less than 8%.

S3. Results

S3.1 VOCs and Non-VOCs

Table S2. Chromatogram information of the VOCs emitted by HTP.

No.	RT (min)	Compound	No.	RT (min)	Compound
1	1.386	Acetone	11	12.057	5-Hydroxymethylfurfural
2	1.475	Methyl acetate	12	12.951	Triacetin
3	1.783	3-Methylfuran	13	13.173	Nicotine
4	2.721	2,5-Dimethylfuran	14	8.577	Formaldehyde[1]
5	3.305	Butyl carbamate	15	11.125	Acetaldehyde[1]
6	3.487	Propylene glycol	16	12.108	Acrolein[1]
7	3.744	2,2-Dimethoxybutane	17	17.461	Propyl Aldehyde[1]
8	9.28	Glycerin	18	24.115	Butanone[1]
9	9.724	4,8-Dimethyltridecane	19	26.696	Benzaldehyde[1]
10	11.361	Pyranone	20	33.106	Tolualdehyde[1]

Note: "1" indicates the HPLC data of VOCs.

Table S3. The chromatograms information of the VOCs emitted by e-cigarettes.

No.	RT (min)	Compound	No.	RT (min)	Compound
1	1.373	Ethanol	10	12.947	Triacetin
2	1.83	Ethyl Acetate	11	13.176	Nicotine
3	3.500	Propylene Glycol	12	13.987	Heptylbutyrolactone
4	9.317	Glycerin	13	14.241	Norpseudoephedrine
5	11.620	Benzoic acid	14	14.561	4-Acetamidobenzaldehyde
6	11.701	DL-Menthol	15	8.115	Formaldehyde[1]
7	12.165	2,3-Dihydroxypropylacetate	16	11.425	Acetaldehyde[1]
8	12.343	Benzylcarbinyl acetate	17	19.325	Crotonaldehyde[1]
9	12.614	N-Ethyl-3-piperidinol	18	24.112	Butanone[1]

Note: "1" indicates the HPLC data of VOCs.

Table S4. The chromatograms information of the VOCs emitted by cigarettes.

No.	RT (min)	Compound	No.	RT (min)	Compound
1	1.216	3-Butynoic acid	50	7.885	Isobutylcyclopentane
2	1.241	Butanal, 3-methyl	51	8.071	2,4-Dimethylhexane
3	1.325	Butanal	52	8.111	2-Methyl-2-cyclopentenone
4	1.381	Acetone	53	8.626	2,3-Pentanedione
5	1.399	Isoprene	54	8.655	2-Nonynoic acid
6	1.471	Methyl acetate	55	8.724	1-Methylcycloheptene
7	1.485	Cyclopentadiene	56	8.782	3-Decyn-2-ol
8	1.545	2-(Z)-pentenol	57	8.995	7-Ethyl-1,3,5-cycloheptatriene
9	1.602	Acetaldol	58	9.079	D-Limonene
10	1.745	5-Methylfuran	59	9.12	1,2,3-Trimethylbenzene
11	1.807	3-Methylfuran	60	9.165	1,2,4-Trimethylbenzene
12	1.856	4-Methyl-3-pentene	61	9.291	Glycerin
13	1.975	2,4-Hexadiene	62	9.392	Phenyl carbamate
14	2.006	1,3,5-Hexatriene	63	9.497	2,3,6-Trimethyl-1,5-heptadiene
15	2.041	1,3-Cyclohexadiene	64	9.587	2,6-Dimethyl 2,6-octadiene
16	2.131	Isobutyraldehyde	65	9.622	1,2,4-Trimethylenecyclohexane
17	2.196	Benzene	66	10.035	Carvomenthen
18	2.241	Acetol	67	10.106	L-Limonene
19	2.281	5-Methyl-1,3-cyclopentadiene	68	10.358	Phenol, 2-methyl
20	2.332	Isopropenyl methyl ketone	69	10.426	Hexahydronaphthalene
21	2.39	1-Nitro-2-propanol	70	10.59	p-Hydroxytoluene
22	2.5	Isopropyl methyl ketone	71	10.826	4-Pyridinol
23	2.624	Ethyl isopropyl ketone	72	11.356	Pyranone
24	2.715	2,5-Dimethylfuran	73	11.471	Benzoic acid
25	2.769	Ethyl propenyl ether	74	11.531	3-Ethylphenol
26	2.835	2,4-Dimethylfuran	75	11.768	Catechol
27	2.99	3-Methylpyridazine	76	11.812	Naphthalene
28	3.03	2-Cyanobutane	77	12.00	4-methyl Benzaldehyde
29	3.114	Methyl pyruvate	78	12.051	5-Hydroxymethylfurfural
30	3.179	Dimethylnitromethane	79	12.421	p-Benzenediol
31	3.299	Butyl carbamate	80	12.789	1-Methylnaphthalene
32	3.421	Propylene glycol	81	12.951	Triacetin
33	3.478	2-Methyl-2-cyclohexen-1-one	82	13.175	Nicotine
34	3.561	Pyrrole	83	13.315	4-Ethylcatecho
35	3.621	2-Methylfuran	84	13.737	2-Hydroxy-6-methylbenzaldehyde
36	3.737	2,2-Dimethoxybutane	85	14.13	Levoglucosan
37	3.902	Toluene	86	14.951	Decanal
38	3.992	Acetohydroximic acid	87	16.379	Neophytadiene
39	4.134	5-Nonynoic acid	88	8.975	Formaldehyde[1]
40	4.42	Heptanal	89	11.347	Acetaldehyde[1]
41	4.603	1,2-Cyclopentanediol	90	11.872	Acrolein[1]
42	4.885	2-Acetylfuran	91	17.709	Propyl Aldehyde[1]
43	5.966	2-Methylpyrazine	92	19.027	Crotonaldehyde[1]
44	6.167	Furfural	93	22.517	Methacrolein[1]
45	6.592	2-Nitrobutanol	94	24.115	Butanone[1]
46	6.636	1,2-Dimethylpropyl acetate	95	26.496	Benzaldehyde[1]
47	7.061	Ethylbenzene	96	30.78	Pentanal[1]
48	7.308	p-Xylene	97	33.106	Tolualdehyde[1]
49	7.816	Styrene			

Note: "1" indicates the HPLC data of VOCs. The boiling points of VOCs measured in this study were between 50 °C and 400 °C.

Table S5. The concentrations of VOCs and non-VOCs measured from HTPs.

HTPs (mg/100 puffs)			
Alkanes		Glycerin[a]	0.171 ± 0.171
2,2-Dimethoxybutane	0.364 ± 0.052	Propylene glycol	2.114 ± 0.101
4,8-Dimethyltridecane[a]	0.031 ± 0.005	Furfurans	
Esters		2-Methylfuran	0.347 ± 0.023
Methyl acetate	0.100 ± 0.016	2,5-Dimethylfuran	0.038 ± 0.038
Triacetin[a]	0.806 ± 0.043	Ketones	
Aldehydes		Pyranone	0.081 ± 0.021
Formaldehyde[1]	0.038 ± 0.003	Acetone	0.806 ± 0.077
Acetaldehyde[1]	0.069 ± 0.009	Butanone[1]	0.034 ± 0.003
Propyl Aldehyde[1]	0.217 ± 0.020	Aromatic Hydrocarbons	
Acrolein[1]	0.241 ± 0.108	Benzaldehyde[1]	0.071 ± 0.005
5-Hydroxymethylfurfural	1.161 ± 0.186	Tolualdehyde[1]	0.116 ± 0.044
Nitrogenous compounds		Non-VOCs	
Nicotine[a]	8.817 ± 0.500	Water	7.070 ± 1.315
Butyl carbamate	0.030 ± 0.030	Tar	4.593 ± 1.225
Alcohols		CO (mg/cig)	0.580 ± 0.018

Note: "1" indicates a component which was analysed by Agilent HPLC system. "a" indicates a VOC boiling point higher than 240 °C (SVOCs). The results are the mean ± SD, n = 3.

Table S6. The concentrations of VOCs and non-VOCs measured from e-cigarettes.

E-cigarettes (mg/100 puffs)			
Esters		Crotonaldehyde[1]	0.021 ± 0.011
Ethyl Acetate	0.101 ± 0.034	Formaldehyde[1]	0.062 ± 0.012
2,3-Dihydroxypropyl acetate[a]	1.253 ± 0.132	Acetaldehyde[1]	0.031 ± 0.006
Benzylcarbinyl acetate	0.061 ± 0.01	Nitrogenous compounds	
Triacetin[a]	4.182 ± 0.395	N-Ethyl-3-piperidinol	32.57 ± 2.92
Heptyl butyrolactone	0.178 ± 0.018	Nicotine[a]	24.63 ± 2.25
Alcohols		Norpseudoephedrine[a]	0.050 ± 0.028
Ethanol	7.701 ± 1.464	4-Acetamidobenzaldehyde[a]	0.176 ± 0.026
Propylene Glycol	168.7 ± 13.8	Aromatic Hydrocarbons	
Glycerin[a]	539.0 ± 51.0	Benzoic acid[a]	15.27 ± 3.65
DL-Menthol	1.466 ± 0.186	Non-VOCs	
Ketones		Water	ND
Butanone[1]	0.013 ± 0.008	Tar	ND
Aldehydes		CO (mg/cig)	ND

Note: "1" indicates the components analysed with Agilent HPLC system. "a" indicates a VOC boiling point higher than 240 °C (SVOCs). The results are the mean ± SD, n = 3.

Table S7. The concentrations of VOCs and non-VOCs measured from cigarettes.

Conventional cigarettes (mg/100 puffs)			
Alkanes		2-Nitrobutanol[a]	0.018 ± 0.012
Isobutylcyclopentane	0.008 ± 0.008	Nicotine[a]	22.94 ± 0.31
2,4-Dimethylhexane	0.035 ± 0.010	4-Pyridinol	0.628 ± 0.080
2,2-Dimethoxybutane	0.496 ± 0.043	Ethers	
1,2,4-Trimethylenecyclohexane	0.091 ± 0.017	Ethyl propenyl ether	0.028 ± 0.014
Hexahydronaphthalene	0.009 ± 0.005	Alkenes	
Esters		Isoprene	5.275 ± 0.895
Methyl acetate	0.219 ± 0.212	Cyclopentadiene	0.343 ± 0.069
1,2-Dimethylpropyl acetate	0.205 ± 0.042	4-Methyl-3-pentene	0.036 ± 0.010
Triacetin[a]	9.299 ± 0.608	2,4-Hexadiene	0.012 ± 0.012
Methyl pyruvate	0.208 ± 0.007	1,3,5-Hexatriene	0.581 ± 0.037
Acids		1,3-Cyclohexadiene	0.086 ± 0.009
3-Butynoic acid	0.020 ± 0.020	5-Methyl-1,3-cyclopentadiene	0.062 ± 0.010
2-Nonynoic acid	0.002 ± 0.002	1-Methylcycloheptene	0.016 ± 0.016
Acetohydroximic acid	0.779 ± 0.298	7-Ethyl-1,3,5-cycloheptatriene	0.013 ± 0.013
5-Nonynoic acid	0.016 ± 0.016	D-Limonene	0.062 ± 0.004
Alcohols		2,3,6-Trimethyl-1,5-heptadiene	0.080 ± 0.014
Acetaldol	0.002 ± 0.002	2,6-Dimethyl-2,6-octadiene	0.112 ± 0.008
Glycerin[a]	5.140 ± 0.546	Neophytadiene[a]	0.891 ± 0.169
2-pentenol	0.060 ± 0.006	L-Limonene	0.987 ± 0.144
Acetol	0.252 ± 0.076	Ketones	
Propylene glycol	0.670 ± 0.043	Acetone	4.48 ± 0.577
3-Decyn-2-ol[a]	0.007 ± 0.007	Isopropenyl methyl ketone	0.220 ± 0.019
1,2-Cyclopentanediol	0.132 ± 0.012	Isopropyl methyl ketone	0.579 ± 0.029
Carveol	0.103 ± 0.010	Ethyl isopropyl ketone	0.357 ± 0.032
Aldehydes		2,3-Pentanedione	0.015 ± 0.007
Formaldehyde[1]	0.155 ± 0.030	2-Methyl-2-cyclohexen-1-one	0.027 ± 0.013
Acetaldehyde[1]	0.131 ± 0.043	2-Methyl-2-cyclopentenone	0.078 ± 0.013
Acrolein[1]	0.925 ± 0.162	Pyranone	0.415 ± 0.035
Propyl Aldehyde[1]	0.429 ± 0.063	Butanone[1]	0.601 ± 0.147
Crotonaldehyde[1]	0.115 ± 0.018	Aromatic Hydrocarbons	
Methacrolein[1]	0.371 ± 0.037	Benzene	0.659 ± 0.094
Pentanal[1]	0.050 ± 0.007	Toluene	1.549 ± 0.202
3-methyl Butanal	0.383 ± 0.383	Ethylbenzene	0.159 ± 0.007
Butanal	0.032 ± 0.016	p-Xylene	0.222 ± 0.043
Isobutyraldehyde	0.574 ± 0.068	Styrene	0.274 ± 0.011
Heptanal	0.014 ± 0.004	1,2,3-Trimethylbenzene	0.109 ± 0.012
5-Hydroxymethylfurfural	3.116 ± 0.168	1,2,4-Trimethylbenzene	0.039 ± 0.003
Furfural	0.446 ± 0.018	Phenol, 2-methyl	0.177 ± 0.021
Decanal	6.561 ± 0.845	p-Hydroxytoluene	0.174 ± 0.027
Levoglucosan	2.022 ± 0.221	Benzoic acid[a]	0.174 ± 0.092
Furfurans		3-Ethylphenol	0.112 ± 0.036
5-Methylfuran	1.762 ± 0.229	Catechol[a]	1.234 ± 0.116
3-Methylfuran	0.030 ± 0.030	4-methyl Benzaldehyde	0.196 ± 0.059
2,5-Dimethylfuran	1.205 ± 0.169	p-Benzenediol[a]	1.551 ± 0.304
2,4-Dimethylfuran	0.024 ± 0.024	4-Ethylcatechol[a]	0.118 ± 0.036
2-Methylfuran	0.077 ± 0.015	2-Hydroxy-6-methylbenzaldehyde	0.235 ± 0.076
2-Acetylfuran	0.057 ± 0.019	Naphthalene	0.026 ± 0.005
Nitrogenous compounds		1-Methylnaphthalene[a]	0.035 ± 0.007
Pyrrole	0.187 ± 0.026	Benzaldehyde[1]	0.020 ± 0.011
Butyl carbamate	0.132 ± 0.036	Tolualdehyde[1]	0.049 ± 0.008
3-Methylpyridazine	0.025 ± 0.002	Non-VOCs	
2-Cyanobutane	0.040 ± 0.003	Water	44.07 ± 6.14
Dimethylnitromethane	0.192 ± 0.054	Tar	110.9 ± 0.8
2-Methylpyrazine	0.025 ± 0.002	CO (mg/cig)	23.24 ± 1.31
Phenyl carbamate[a]	0.217 ± 0.010		

1-Nitro-2-propanol	0.177 ± 0.019

Note: "1" indicates the components analysed with the Agilent HPLC system. "a" indicates a VOC boiling point higher than 240 °C (SVOCs). The results are the mean ± SD, $n = 3$.

S 3.2 Human health risk characterisation for different types of cigarettes

Table S8. The related parameters of the risk assessment.

Aldehydes/ketones	RFC_i [a] mg/m^3	IUR_i[a] (μg/m^3)$^{-1}$	RFD_i mg/kg-day	SF_i (mg/kg-day)$^{-1}$	mmHg [b]	Vapor pressure (Pa)	RR[c] (%)
Formaldehyde	9×10^{-3}	1.3×10^{-5}	2.3×10^{-3}	0.0509	3890	518624	98.61 %
Acetaldehyde	9×10^{-3}	2.2×10^{-6}	2.3×10^{-3}	0.0086	902	120256	97.98 %
Acrolein	2×10^{-5}	/	5.1×10^{-6}	/	274	36530	97.46 %
Propyl Aldehyde	8×10^{-3}	/	2.0×10-3	/	317	42263	97.53 %
Butanone	5	/	1.28	/	90.6	12079	96.98 %
Benzene	3×10^{-2}	2.2×10^{-6}	7.6×10-3	0.0086	94.8	12638.9	97.00 %
Toluene	5	/	1.28	/	28.4	3786	96.48 %
Ethylbenzene	1	2.5×10^{-6}	0.255	0.0098	9.6	1279.8	96.01 %
p-Xylene	1×10^{-1}	/	0.0255	/	8.8	1178.5	95.97 %
Styrene	1	/	0.255	/	6.4	853.2	95.83 %
1,2,3-Trimethylbenzene	2×10^{-1}	/	5.11×10-3	/	1.69	225.3	95.25 %
1,2,4-Trimethylbenzene	2×10^{-1}	/	5.11×10-3	/	2.1	279.9	95.35 %
Naphthalene	3×10^{-3}	3.4×10^{-5}	0.7658	0.1332	8.5×10^{-2}	11.33	93.95%
Acetone	3×10	/	7.66	/	232	30930	97.39 %
Crotonaldehyde	1×10-2	/	2.5×10-3	/	30.8	4000	96.50 %
Catechol	1.4×10^{-1}	/	0.00357	/	3.6×10^{-3}	0.487	92.59 %
p-Benzenediol	8.8×10^{-2}	/	0.0204	/	2.39×10^{-4}	0.0032	90.41 %
Furfural	5×10^{-2}	/	0.00128	/	2.2	294.6	95.37 %
Heptanal	3×10^{-3}	/	7.6×10-4	/	3.52	469.29	95.57 %
Isoprene	/	2.2×10^{-8}	/	8.6×10^{-5}	550	73327	97.77 %
CO	2.3×10	/	2.3×10-3	/	1.5×10^{8}	2.06×10^{10}	103.21 %

Note: "a" indicates the values of RfC_i and IUR_i obtained by from USEPA, CalEPA, ATSDR, etc. [9–12]. RfC_i is the reference concentration of constituent i for non-cancer, and IUR_i is the inhalation unit risk of constituent i for cancer (μg/m^3)$^{-1}$; "b" represents the value gained from http://www.chemspider.com/.

The reference dose (*RFD*) and carcinogenic slope factor (*SF*) of constituent i were calculated by an extrapolation model presented in Equations (1) and (2) [13]:

$$RFD_i = RFC_i \times DAIR_a / BW_a \quad (1)$$

$$SF_i = IUR_i \times BW_a / DAIR_a \quad (2)$$

where RFD_i is a reference dose of constituent *i* for non-cancer, mg/kg-day; SF_i is the carcinogenic slope factor of constituent *i* for cancer, (mg/kg-day)$^{-1}$; *DAIRa* is the adult daily air inhalation rate (m^3/d; recommended value is 14.5 m^3/d) [13]; *BWa* is the adult body weight (kg; recommended value is 56.8 kg) [13].

Table S9. The value of U and CDI for different constituents, *i*, emitted by different types of cigarettes.

	U			*CDIi* (non-carcinogenic)			*CDIi* (carcinogenic)		
Constituents	HTPs	E-C	C-C	HTPs	E-C	C-C	HTPs	E-C	C-C
Formaldehyde	2.6×10^{-4}	4.3×10^{-4}	1.1×10^{-3}	1.99×10^{-3}	1.16×10^{-3}	3.11×10^{-3}	1.49×10^{-3}	8.70×10^{-4}	2.33×10^{-3}
Acetaldehyde	4.7×10^{-4}	2.1×10^{-4}	9.0×10^{-4}	3.59×10^{-3}	5.76×10^{-4}	2.61×10^{-3}	2.69×10^{-3}	4.32×10^{-4}	1.96×10^{-3}
Acrolein	1.6×10^{-3}	/	6.3×10^{-3}	1.25×10^{-2}	/	1.84×10^{-2}	9.35×10^{-3}	/	1.38×10^{-2}
Propyl Aldehyde	1.5×10^{-3}	/	2.9×10^{-3}	1.12×10^{-2}	/	8.52×10^{-3}	8.42×10^{-3}	/	6.39×10^{-3}
Butanone	2.3×10^{-4}	/	4.1×10^{-3}	1.75×10^{-3}	2.39×10^{-4}	1.19×10^{-2}	1.31×10^{-3}	1.79×10^{-4}	8.90×10^{-3}
Benzene	/	/	4.5×10^{-3}	/	/	1.30×10^{-2}	/	/	9.76×10^{-3}
Toluene	/	/	1.0×10^{-2}	/	/	3.04×10^{-2}	/	/	2.28×10^{-2}
Ethylbenzene	/	/	1.1×10^{-3}	/	/	3.11×10^{-3}	/	/	2.33×10^{-3}
p-Xylene	/	/	1.5×10^{-3}	/	/	4.34×10^{-3}	/	/	3.25×10^{-3}
Styrene	/	/	1.8×10^{-3}	/	/	5.35×10^{-3}	/	/	4.01×10^{-3}
1,2,3-Trimethylbenzene	/	/	7.3×10^{-4}	/	/	2.11×10^{-3}	/	/	1.59×10^{-3}
1,2,4-Trimethylbenzene	/	/	2.6×10^{-4}	/	/	7.57×10^{-4}	/	/	5.68×10^{-4}
Naphthalene	/	/	1.7×10^{-4}	/	/	4.97×10^{-4}	/	/	3.73×10^{-4}
Acetone	5.5×10^{-3}	/	3.1×10^{-2}	4.17×10^{-2}	/	8.88×10^{-2}	3.12×10^{-2}	/	6.66×10^{-2}
Crotonaldehyde	/	/	7.8×10^{-4}		3.84×10^{-4}	2.26×10^{-3}	/	2.88×10^{-4}	1.69×10^{-3}
Catechol	/	/	8.0×10^{-3}	/	/	2.33×10^{-2}	/	/	1.74×10^{-2}
p-Benzenediol	/	/	9.8×10^{-3}	/	/	2.86×10^{-3}	/	/	2.14×10^{-2}
Furfural	/	/	3.0×10^{-3}	/	/	8.66×10^{-3}	/	/	6.50×10^{-3}
Heptanal	/	/	9.4×10^{-5}	/	/	2.72×10^{-4}	/	/	2.04×10^{-4}
Isoprene	/	/	3.5×10^{-2}	/	/	1.05×10^{-1}	/	/	7.88×10^{-2}
CO	0.035	/	1.399	2.65×10^{-1}	/	4.07	1.99×10^{-1}	/	3.05

Note: “E-C” indicates e-cigarettes; “C-C” indicates conventional cigarettes.

References

1. Flora, J.W.; Meruva, N.; Huang, C.B.; Wilkinson, C.T.; Ballentine, R. Characterization of potential impurities and degradation products in electronic cigarette formulations and aerosols. *Regul. Toxicol. Pharmacol.* **2016**, *74*, 1–11. https://doi.org/10.1016/j.yrtph.2015.11.009.
2. Wagner, K.A.; Flora, J.W.; Melvin, M.S.; Avery, K.C.; Ballentine, R.M.; Brown, A.P.; McKinney, W.J. An evaluation of electronic cigarette formulations and aerosols for harmful and potentially harmful constituents (HPHCs) typically derived from combustion. Regul. Toxicol. Pharmacol. 2018, 95, 153–160. https://doi.org/10.1016/j.yrtph.2018.03.012.
3. CORESTA. Recommended Method No 70. Determination of selected volatile organic compounds in the mainstream smoke of cigarettes-gas chromatography-mass spectrometry method. CORESTA. 2014. https://www.coresta.org/search/site/CRM%2520No.70 (accessed on 3/1/2021).
4. GB/T 27523-2011. Cigarettes-Determination of volatile organic compounds (1,3-butadiene, isoprene, acrylonitrile, benzene, toluene) in mainstream smoke-GC-MS method. General Administration of Quality Supervision, Inspection and Quarantine of the People's Republic of China. 2011. https://www.doc88.com/p-701862311399.html (accessed on 5/6/2021).
5. CORESTA. Recommended method N^0. 74. Determination of selected carbonyls in mainstream cigarette smoke by HPLC. CORESTA.2018.https://www.coresta.org/search/site/Determination%20of%20selected%20carbonyls%20in%20main-stream%20cigarette%20smoke%20by%20HPLC (accessed on 3/1/2021).
6. GB/T19609-2004. Cigarette-Determination of total and nicotine-free dry particulate using a routine analytical smoking machine. General Administration of Quality Supervision, Inspection and Quarantine of the People's Republic of China.2004. https://www.doc88.com/p-2562493560107.html (accessed on 5/6/2021).
7. GB/T 23203.1-2013. Cigarettes-Determination of water in smoke condensates-Part 1: Gas-chromatographic. General Administration of Quality Supervision, Inspection and Quarantine of the People's Republic of China. 2013. https://www.doc88.com/p-6943506288379.html (accessed on 5/4/2021).
8. CORESTA. Recommended Method No 55. Determination of Carbon Monoxide in the Vapour Phase of Cigarette Sidestream Smoke using a Fishtail Chimney and a Routine Analytical/Linear Smoking Machine. CORESTA. 2011. https://www.coresta.org/search/site/CRM%20No.55 (accessed on 8/8/2021).
9. U.S EPA. The Integrated Risk Information System. 1985. Available online: https://cfpub.epa.gov/ncea/iris/search/index.cfm (accessed on 5/1/2021).
10. Pack, E.C.; Kim, H.S.; Jang, D.Y.; Koo, Y.J.; Yu, H.H.; Lee, S.H.; Lim, K.M.; Choi, D.W. Risk assessment of toxicants on WHO TobReg priority list in mainstream cigarette smoke using human-smoked yields of Korean smokers. *Environ. Res.* **2019**, *169*, 206–219. https://doi.org/10.1016/j.envres.2018.11.012.
11. Xie, J.; Marano, K.M.; Wilson, C.L.; Liu, H.; Gan, H.; Xie, F.; Naufal, Z.S. A probabilistic risk assessment approach used to prioritize chemical constituents in mainstream smoke of cigarettes sold in China. *Regul. Toxicol. Pharmacol.* **2012**, *62*, 355–362. https://doi.org/10.1016/j.yrtph.2011.10.017.
12. Liang, W. Volatile organic compounds, odor, and inhalation health risks during interior construction of a fully furnished residential unit in Nanjing, China. *Build. Environ.* **2020**, *186*, 107366.
13. HJ 25.3-2014. Technical guidelines for risk assessment of contaminated sites. China Environmental Press. **2014**, 1–56. https://www.docin.com/p-2215481867.html (accessed on 5/7/2021).
